# Pericardial tamponade and coexisting pulmonary embolism as first manifestation of non-advanced lung adenocarcinoma

**DOI:** 10.11604/pamj.2014.18.15.2469

**Published:** 2014-05-03

**Authors:** Salwa Akhbour, Brahim Amine Khennine, Latifa Oukerraj, Jamila Zarzur, Mohamed Cherti

**Affiliations:** 1 Cardiology B department, CHU Ibn Sina, faculty of medicine and pharmacy, university Mohammed V Souissi, Rabat, Morocco

**Keywords:** Pericardial tamponade, pulmonary embolism, lung adenocarcinoma

## Abstract

Pericardial effusion and pulmonary embolism are relatively common complications of malignancy and are uncommon as its initial manifestation. This report describes a case of a patient, who presented with this association, due to an underlying pulmonary adenocarcinoma. When a major pericardial effusion is associated with pulmonary hypertension, some echocardiographic signs may redress the diagnosis. This case emphasizes a challenge diagnostic which may be guided by high right ventricular pressure and on the other hand the importance of keeping both these conditions in mind when dealing with context of malignancy.

## Introduction

Pericardial effusion is commonly found in patients with metastatic malignant disease. Although the leading cause is lung cancer, it is also linked to breast cancer, leukemia and lymphoma [1]. Pericardial tamponade in patients with malignancies is rarely seen as an initial presenting symptom, and its association with pulmonary embolism is even less common. To our knowledge, this is the fourth case of pericardial tamponade in association with pulmonary embolism due to an underlying lung adenocarcinoma as an initial symptom [2–4].

## Patient and observation

A 63-year-old woman was referred to our intensive care unit for acute pericarditis. The patient had no history of smoking, systemic diseases or cancer. Five days prior to her referral, she had been admitted to another hospital for acute pericarditis with an echocardiogram that showed a pericardial effusion, and 200ml of non-coagulable bloody fluid was removed. Her symptoms had reportedly started four weeks earlier, when she began to complain of weakness, progressive dyspnoea and chest pain.

**Figure 1:**
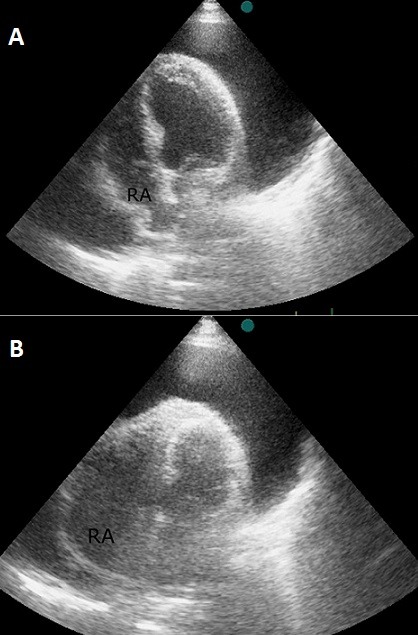
Transthoracic echocardiography (apical 4-chamber View) Large pericardial effusion with right atrial diastolic collapse (A) which missing at systole (B). Note the absence of right ventricular collapse. RA: right atrium

**Figure 2:**
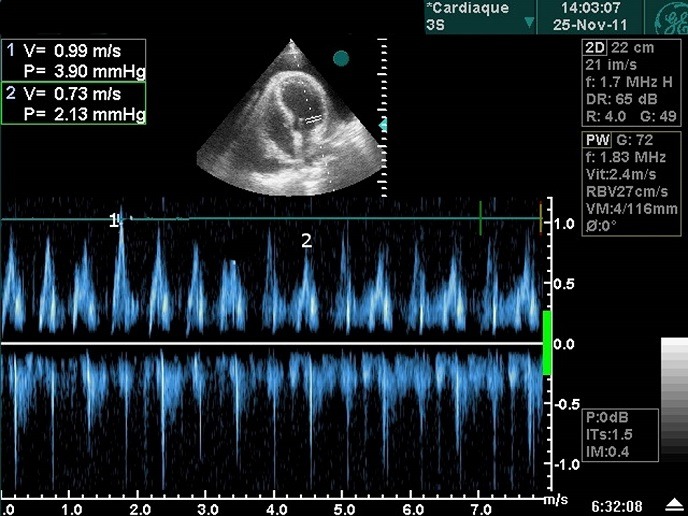
Respiratory variation in mitral flow velocities >25%

She was confused when she arrived in our department. A cursory physical examination revealed a congested jugular and oedema in the lower extremities. Blood pressure was low (80/60 mmHg), with an increased heart rate (110/min) and distant heart sounds without a pericardial rub or paradoxical pulse. The electrocardiogram showed prominent low voltage QRS complexes in all derivations. Two-dimensional echocardiography showed a large pericardial effusion and marked diastolic collapse of the right auricular (Figure 1) with significant respiratory variation in mitral flow velocity (Figure 2), there was no collapse of the right ventricule.

Emergency pericardiocentesis was performed via a subxiphoid approach. After the removal of 350ml of non-coagulable bloody fluid, clinical symptoms rapidly improved and her blood pressure rose to 100/60mmHg. A post pericardiocentesis echocardiography showed a high pulmonary artery systolic pressure with pulmonary artery systolic pressure at 50 mmHg.

A chest radiograph revealed an enlarged cardiac silhouette, bilateral pulmonary interstitial infiltrates, and a small bilateral pleural effusion.

Laboratory results showed an elevated erythrocyte sedimentation rate of 50mm/h, protein C reactive at 130 mg/l, and a white cell count of 12,000/mm^3^. Cancer antigen 19-9 (CA), CA 125 and CA15-3 analysis revealed very elevated levels. Systemic diseases and tuberculosis were excluded.

**Figure 3:**
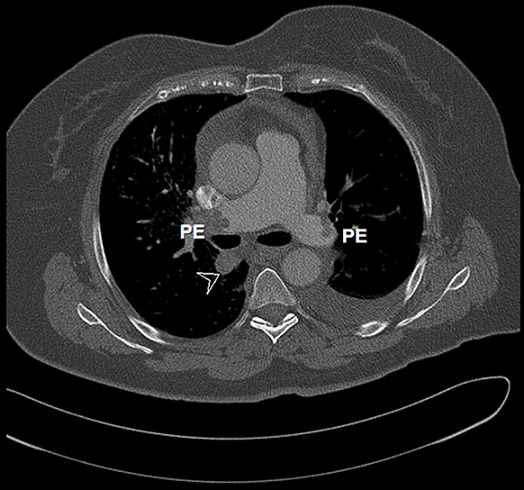
A chest computed tomography scan showing mass lesion in the right lower lung lobe (arrow), and bilateral proximal pulmonary embolism (PE)

A computed tomography scan was performed to identify the origin of pulmonary hypertension, it revealed a mass lesion in the right lower lung lobe (2.1cm), with pleural effusion and bilateral proximal pulmonary embolism (Figure 3). A lung mass biopsy with histo-pathological examination revealed primary moderate differentiated adenocarcinoma. The patient received anticoagulation therapy and was referred to the department of oncology.

## Discussion

Pericardial effusion is a well-known complication of many advanced malignancies such as lung cancer, breast cancer, lymphomas and leukemias [1]. Although infrequent, symptomatic pericardial effusion with hemodynamic compromise can be the initial presentation of underlying malignancies [5]. It can occur because of accumulated fluid amounts as low as 150 ml if the accumulation occurs rapidly. On the other hand, the slow accumulation of fluid, in some instances as much as two liters, may not cause tamponade. Cardiac involvement can be related to retrograde lymphatic, hematogenic, direct, or transvenous extension [6].

Paradoxical pulse is an important clinical finding that should direct attention to the possibility of cardiac tamponade. The predominant mechanism responsible for paradoxical pulse in cardiac tamponade is an exaggeration of the normal inspiratory increase in right ventricular filling that impedes left ventricular filling by causing transient leftward displacement of the interventricular septum [4]. Moderate right ventricular pressure overload can obscure this sign.

Echocardiography Doppler is a portable, non-invasive imaging modality that was ideally suited in this instance to rapidly confirm the clinical suspicion of tamponade and help guiding therapeutic management [7]. It revealed pericardial effusion with right atrial and ventricular compression at diastole. The lack of right ventricular collapse due to high right ventricular pressure in our patient should merit testing for pulmonary hypertension [2].

In the absence of coexistent chronic obstructive pulmonary diseases or other conditions causing pulmonary hypertension, the diagnosis of pulmonary embolism may be suspected only when left-sided pressures fall while right-sided pressures remain steady after successful pericardiocentesis [8].

Pericardial effusion and pulmonary embolism associated with hyper-coagulable states are relatively common complications of malignancy. Usually, their presence is easy to detect when isolated. Their simultaneous presentation, as in our case, is extremely rare. This association is a diagnostic challenge because each can cause chest pain, tachycardia, hypotension, and signs of right heart failure or systemic congestion at presentation. Management may also be challenging because of relative contraindication to anticoagulation in the presence of pericardial lesion.

## Conclusion

We believe that it is necessary to consider a possible diagnosis of pericardial tamponade of various causes, even advanced malignancies, in otherwise healthy patients admitted with the aforementioned symptoms. Cardiac tamponade and pulmonary embolism are a rare but potentially fatal cancer complication. Their coexistence may have paradoxically saved our patient's life, as the raised right ventricular pressure created by the pulmonary emboli delayed the onset of cardiac tamponade.
